# Electrical stimulation directs engineered cardiac tissue to an age-matched native phenotype

**DOI:** 10.1177/2041731412455354

**Published:** 2012-07-27

**Authors:** Richard A Lasher, Aric Q Pahnke, Jeffrey M Johnson, Frank B Sachse, Robert W Hitchcock

**Affiliations:** 1 Department of Bioengineering, University of Utah, Salt Lake City, UT, USA; 2 Nora Eccles Harrison Cardiovascular Research and Training Institute, University of Utah, Salt Lake City, UT, USA

**Keywords:** Tissue engineering, confocal microscopy, structural modeling, cardiac muscle, cardiac cell

## Abstract

Quantifying structural features of native myocardium in engineered tissue is essential for creating functional tissue that can serve as a surrogate for in vitro testing or the eventual replacement of diseased or injured myocardium. We applied three-dimensional confocal imaging and image analysis to quantitatively describe the features of native and engineered cardiac tissue. Quantitative analysis methods were developed and applied to test the hypothesis that environmental cues direct engineered tissue toward a phenotype resembling that of age-matched native myocardium. The analytical approach was applied to engineered cardiac tissue with and without the application of electrical stimulation as well as to age-matched and adult native tissue. Individual myocytes were segmented from confocal image stacks and assigned a coordinate system from which measures of cell geometry and connexin-43 spatial distribution were calculated. The data were collected from 9 nonstimulated and 12 electrically stimulated engineered tissue constructs and 5 postnatal day 12 and 7 adult hearts. The myocyte volume fraction was nearly double in stimulated engineered tissue compared to nonstimulated engineered tissue (0.34 ± 0.14 vs 0.18 ± 0.06) but less than half of the native postnatal day 12 (0.90 ± 0.06) and adult (0.91 ± 0.04) myocardium. The myocytes under electrical stimulation were more elongated compared to nonstimulated myocytes and exhibited similar lengths, widths, and heights as in age-matched myocardium. Furthermore, the percentage of connexin-43-positive membrane staining was similar in the electrically stimulated, postnatal day 12, and adult myocytes, whereas it was significantly lower in the nonstimulated myocytes. Connexin-43 was found to be primarily located at cell ends for adult myocytes and irregularly but densely clustered over the membranes of nonstimulated, stimulated, and postnatal day 12 myocytes. These findings support our hypothesis and reveal that the application of environmental cues produces tissue with structural features more representative of age-matched native myocardium than adult myocardium. We suggest that the presented approach can be applied to quantitatively characterize developmental processes and mechanisms in engineered tissue.

## Introduction

Establishing hallmarks of the native myocardium in engineered tissue is essential for creating functional tissue that can serve as a surrogate for in vitro testing or the eventual replacement of diseased or injured myocardium.^1^ Quantitative measures of structural and functional tissue characteristics form a technical cornerstone for the development and testing of engineered cardiac tissue. Native tissue is complex and exhibits a three-dimensional (3D) multicellular structure and function. This 3D microenvironment has profound effects on the properties, behaviors, and functions of resident cells.^1–3^ Furthermore, native tissue exhibits astonishing variation in the quantity, density, and morphology of cardiac cells during development, among species, between tissue types and in disease states.^4–6^ Most engineered cardiac tissue aims to replicate left ventricular myocardium, which is heterogeneous and composed of densely packed myocytes, fibroblasts, and other cell types.

Fibroblasts account for the majority of the cells in the heart and play important roles in normal cardiac function and disease.^7,8^ Although myocytes only account for 20%-40% of the cells that make up cardiac tissue, they occupy approximately 80%-90% of the tissue volume and are the contractile cells solely responsible for pump function.^9,10^ Alterations in myocyte geometry and structure are known to occur during development and in disease states.^11–13^ Myocyte structures that are critical for cardiac function include sarcomeres and gap junctions. Sarcomeres, the fundamental unit of contraction, occupy a large fraction of the intracellular volume and are highly aligned in healthy myocytes. Gap junctions allow for rapid electrical signaling between myocytes necessary for synchronous cardiac contraction. Connexin-43 (Cx43), the predominant isoform of gap junction channels in ventricular myocytes,^14,15^ has a half-life of 2 h. The continuous turnover allows Cx43 to redistribute along the cell surface in response to environmental conditions.^16,17^ The distribution of Cx43 is known to vary during development and in disease states.^18,19^ For example, in rat cardiac tissue, Cx43 redistributes in response to tissue maturity. In neonatal tissue, Cx43 clusters are found to be distributed over the myocyte membrane. As the tissue matures, Cx43 slowly becomes organized and at approximately 90 days after birth concentrates at the cell ends (i.e. polarized).^18^ Gap junctions also remodel due to disease. For example, as human cardiac hypertrophy progresses into heart failure, Cx43 expression decreases and accumulates at the lateral sides of the myocytes instead of the ends (i.e. lateralized).^4,14,20^ Gap junctions can be coerced to rearrange in vitro. A recent study in 2D monolayers of neonatal rat myocytes indicated polarization of Cx43 localization by stretching.^21^ The functional importance and dynamic nature of Cx43 makes it a target for analysis, and these types of responses may indicate some level of control over engineered cardiac tissue.

Several approaches have been developed to produce 3D engineered cardiac tissue, including seeding preformed scaffold materials with cells,^22^ entrapping cells in a 3D environment,^23^ stacking cell sheets,^24^ and decellularizing and recellularizing tissue^25^ (reviewed in detail in Refs^26,27^). The application of electrical stimulation,^22,28,29^ mechanical stimulation,^30–32^ or perfusion^33^ has been shown to aid in the tissue development. To investigate the structure of these engineered tissues, most reported methods rely on qualitative interpretation of the 2D images. A more comprehensive analysis of structure can be accomplished through 3D confocal microscopy.^34,35^ Confocal microscopy is based on fluorescent labeling and has the ability to control the depth of field (slice resolution of <1 µm), reject out-of-focus light, and collect sequential optical sections from thick specimens.^36,37^ The application of 3D confocal imaging to quantitatively characterize structure has not been widely performed on engineered tissue.

The hypothesis of this study is that the application of environmental cues directs engineered tissue toward a phenotype resembling that of age-matched native myocardium. The hypothesis was tested by applying 3D confocal imaging and image analysis to characterize hallmarks of cardiac tissue, including myocyte geometry and spatial distribution of Cx43, in engineered cardiac tissue with and without the application of electrical stimulation. The results of the study support our hypothesis and reveal that the application of environmental cues produces tissue with structural features resembling age-matched native myocardium as opposed to adult tissue.

## Methods

### Cell isolation

All animal procedures were performed in accordance with an approved protocol by the University of Utah Institutional Animal Use and Care Committee. Ventricular cardiac cells were harvested from 1-day old Sprague Dawley rats (Charles River, MA) using a protocol and supplies from Worthington Biochemical (Lakewood, NJ). Briefly, hearts were aseptically removed and collected in calcium- and magnesium-free Hank’s balanced salt solution. Atria were removed, and the ventricles were finely minced and digested in 50 µg/mL trypsin at 4°C overnight. Further digestion was performed the following day with collagenase (1500 units) in Leibovitz L-15 media. Cell suspensions were triturated, filtered, centrifuged, and resuspended in culture medium. Culture medium was made following the methods described by Hansen et al.^38^ using Dulbecco’s modified Eagle’s medium (DMEM) F12 (Thermo Fisher Scientific, Waltham, MA), 10% equine serum (Thermo Fisher Scientific), 2% chick embryo extract (Gemini Bioproducts, West Sacramento, CA), 50 µg/mL human insulin (Sigma–Aldrich, St. Louis, MO), 2 mM l-glutamine (Thermo Fisher Scientific), 20 U/mL penicillin (MP Biomedicals, Solon, OH), 50 µg/mL streptomycin (MP Biomedicals), 63 µg/mL tranexamic acid (Sigma–Aldrich), and 33 µg/mL aprotinin (Sigma–Aldrich).

### Sample preparation and culture

**Figure 1.**
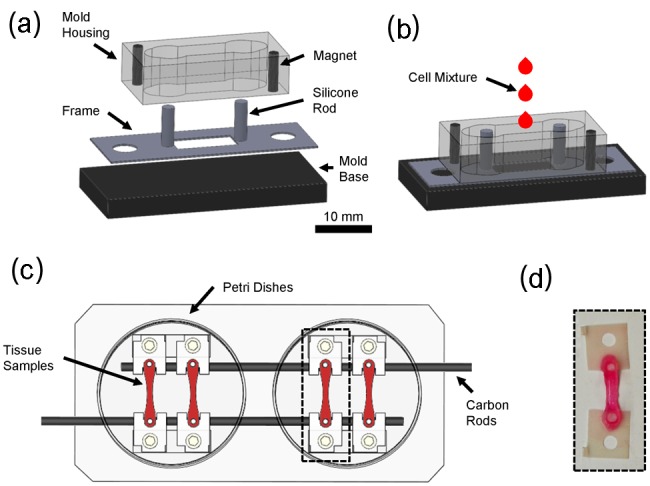
Sample preparation and bioreactor. (a) Exploded and (b) assembled view of mold for producing tissue samples, (c) bioreactor consisted of two Petri dishes and carbon rods for electrical stimulation, and (d) tissue sample.

Fibrin-based engineered tissue samples were fabricated using methods described by Hansen et al.^38^ Briefly, a reconstitution mixture was prepared on ice comprising of 4.1 × 10^6^ cells/mL, 5 mg/mL bovine fibrinogen (Sigma–Aldrich), and 100 µL/mL Matrigel (BD Biosciences, San Jose, CA). For each sample, 485 µL of reconstitution mixture was mixed with 15 µL thrombin (100 U/mL; Sigma–Aldrich) and transferred to a custom mold (Figure 1). The custom mold was contained in a Petri dish and consisted of a Delrin^®^ (McMaster-Carr, Los Angeles, CA) housing and base, each containing two neodymium magnets (Applied Magnets, Plano, TX), which allowed for easy coupling and uncoupling of the mold and base. The housing had a center channel 4.8 mm in width and 20 mm in length with 6.35 mm holes centered with the silicone posts, and contained cylinder-shaped (1.6 mm diameter × 6.4 mm length) magnets. The base was 34 mm × 20 mm and contained disk-shaped (4.8 mm diameter × 1.6 mm thick) magnets that aligned with the housing. Rectangular frames (34 × 12 mm) were cut from 0.30-mm-thick polyester sheets (Mylar^®^; Fralock, Valencia, CA) using a cutting plotter (Graphtech FC7000, Irvine, CA) and AutoCAD (San Rafael, CA), and sandwiched between the housing and base. Frames had a rectangular center (10 mm × 4.8 mm) and two 4-mm through holes spaced 26 mm apart (center to center). Silicone rods (2 mm diameter × 7 mm length) were fabricated from a platinum-cured silicone elastomer (VST-50; Factor II, Lakeside, AZ) and attached to either side of the frame window (spaced 12 mm center to center). The silicone posts served to suspend the fibrin-based gel (Figure 1(d)). Samples were allowed to polymerize at 37°C for 90 min. After 30 min of polymerization, 500 µL of the culture medium was added to keep the sample hydrated and to aid in removal of the mold from the tissue sample. The frame was cut on both sides, and the sample was elongated by 40% and secured with nylon screws into a custom bioreactor consisted of two Petri dishes outfitted with carbon rods spaced 2 cm apart for electrical stimulation (Figure 1(c)).^39^

Engineered tissue samples were precultured for 3 days before onset of electrical stimulation.^22^ Following preculture, samples were subjected to electrical field stimulation (2 ms symmetric biphasic square pulses, 4 V/cm, 1 Hz) for 9 days. Nonstimulated samples served as controls for stimulated samples. Bright field images of central regions of the engineered tissue samples were obtained at days 3, 6, 9, and 12 of culture. The diameter was measured, and the cross-sectional area was estimated assuming a cylindrical cross section. The percent decrease in sample size was calculated normalized to the start of the stimulation, that is, day 3 of culture. At the end of culture, samples were fixed with 4% paraformaldehyde and stored in phosphate-buffered saline (PBS) at 4°C.

### Excitation threshold and maximum capture rate

The excitation threshold (ET) and maximum capture rate (MCR) were measured at days 6, 9, and 12 of culture and for postnatal day 3 (P3) rat hearts following methods described previously.^29,33^ ET was defined as the minimum voltage required to elicit synchronous contractions over the entire sample and MCR as the maximum frequency for synchronous contractions at 150% of the ET. For engineered tissue samples, measurements were made following 30 min of media exchange. For P3 hearts, rats (n = 4) were anesthetized with isofluorane inhalation. Following thoracotomy, hearts were quickly excised and placed in a modified oxygenated Tyrode’s solution (126 mM NaCl, 11 mM dextrose, 0.1 mM CaCl_2_, 13.2 mM KCl, 1 mM MgCl_2_, 12.9 mM NaOH, and 24 mM 4-(2-hydroxyethyl)-1-piperazineethanesulfonic acid (HEPES)) at room temperature. Strips of left ventricular myocardium (≈2 mm × 2 mm × 4 mm) were excised and placed in the same bioreactors used for tissue culture. For all samples, ET was measured by applying square-wave monophasic pulses of 2 ms starting at 0 V/cm and then increasing until the sample was observed to beat synchronously. MCR was measured by setting the voltage to 150% of the ET and increasing the frequency until the contractions became asynchronous, irregular, or ceased.

### Native tissue preparation and sectioning

P12 and adult rat hearts were used for comparison to the engineered tissue samples. Tissue was processed as previously described.^35^ Briefly, rats were anesthetized through methoxyflurane, and hearts were quickly removed. Hearts were perfused with a zero calcium Tyrode’s solution for 5 min followed by 2% paraformaldehyde for 15 min for fixation using the retrograde Langendorff method.^40^ Whole hearts and engineered tissue samples were stored in 30% sucrose in preparation for sectioning. For adult hearts, biopsies were performed with a 5-mm-diameter biopsy punch through the left ventricular wall. P12 hearts were maintained as whole hearts. The biopsied adult hearts, whole P12 hearts, and engineered tissue samples were frozen in tissue freezing medium (Triangle Biomedical Sciences, Durham, NC) and sectioned using a cryostat (Leica CM1950; Wetzlar, Germany). For adult heart biopsies, tissues were sectioned parallel to the epicardial surface and for P12 hearts from the top of the ventricles to approximately 2 mm from the apex to produce 80- to 100-µm-thick sections. Longitudinal and transverse cross sections with a thickness of 100 µm were produced for engineered tissue samples.

### Fluorescent labeling

Fluorescent labeling was performed before sectioning for engineered tissue and after sectioning for native tissue samples. Tissue samples were labeled as described previously.^35^ Samples were either quad-labeled with wheat germ agglutinin (WGA) to identify cell borders, α-sarcomeric actinin to identify myocytes, Cx43 to identify gap junction channels, and 4′,6-diamidino-2-phenylindole dihydrochloride (DAPI) to identify nuclei or tri-labeled with α-sarcomeric actinin to identify myocytes, vimentin to identify nonmyocytes (mostly fibroblasts), and with DAPI to identify nuclei.

All labeling was performed on a laboratory platform rocker at room temperature (Thermo Fisher Scientific). The antibodies were diluted in blocking solution consisting of 4% goat serum (Invitrogen, Carlsbad, CA) and 0.5% Triton X-100 (Fisher Scientific, Pittsburgh, PA) diluted in PBS. Rinsing was performed between all incubation steps and included three 15-min rinses. For quad-labeling, samples were incubated for 16 h with WGA-conjugated CF488 (20–40 µg/mL in PBS; 29022; Biotium, Hayward, CA), 16 h with mouse IgG_1_ anti-α-sarcomeric actinin (1:100; ab9465; Abcam, Cambridge, MA) followed by 6 h with goat anti-mouse IgG_1_-conjugated Alexa Fluor 633 (1:200; A21126; Invitrogen), 1 h with Image-iT^®^ FX signal enhancer (Alexa Fluor 555 Goat Anti-Rabbit SFX Kit, A31630; Invitrogen) to block nonspecific antibody binding, 16 h with rabbit anti-GJA1 (1:100; SAB4300504; Sigma–Aldrich) followed by 6 h with goat anti-rabbit IgG-conjugated Alexa Fluor 555 (1:200; A31630; Invitrogen), and 3 h with DAPI (1:500; Sigma–Aldrich). For tri-labeling, samples were incubated for 16 h with mouse IgG_1_ anti-α-sarcomeric actinin (1:100; ab9465; Abcam) followed by 6 h with goat anti-mouse IgG_1_-conjugated Alexa Fluor 633 (1:200; A21126; Invitrogen), 16 h with mouse monoclonal anti-vimentin-conjugated Cy3 (1:50; C9080; Sigma–Aldrich), and 3 h with DAPI (1:500; Sigma–Aldrich). Tissue samples were stored in PBS.

### Confocal imaging

The 3D image stacks were acquired for samples labeled with WGA, α-sarcomeric actinin, Cx43, and DAPI on a Zeiss LSM 5 Duo confocal microscope (Carl Zeiss, Jena, Germany) using a 40× oil-immersion objective lens with a numerical aperture of 1.3.^35^ The sectioned tissue samples were placed on a glass slide and surrounded by 15–30 µL of Fluoromount-G^™^ Slide Mounting Medium (Electron Microscopy Sciences, Hatfield, PA). The tissue sample was covered with a coverslip (no. 0) and placed on the imaging stage. The *x*-axis of the image stack was aligned with the long axis of the myocytes by visual inspection and adjustment of the scan direction. For engineered tissue samples, sections were briefly scanned using a 10× objective lens to locate dense regions of myocytes. Only regions with high cell density were imaged in this study.

The image stacks were acquired with a voxel size of 200 nm × 200 nm × 200 nm and a typical field of view of 1024 × 768 × 200 voxels using a multitrack protocol for quasi-simultaneous imaging of fluorophores in each 2D image slice. Laser lines with a wavelength of 364, 488, 543, and 633 nm were alternately applied to excite their associated fluorophores and collected using long-pass 385 nm, band-pass 505–555 nm, long-pass 560 nm, and band-pass 630–650 nm filters, respectively. The dwell time was typically 1.3–1.5 µs/pixel resulting in a total imaging time of approximately 1 h per image stack. Signal-to-noise ratio (SNR) of each image stack was measured as described previously.^35^ The image stacks with a SNR below 3 were rejected. For whole sample examination of engineered tissue, 2D images were acquired using a 10× objective of central transverse and longitudinal tissue sections stained with α-sarcomeric actinin, vimentin, and DAPI. Higher magnification (40×) 2D images were also acquired for engineered and native tissue samples stained with α-sarcomeric actinin, vimentin, and DAPI.

### Image processing

The image stacks were processed to improve image quality as previously described.^34,35^ In brief, the image stacks were processed to remove background, correct for depth-dependent attenuation, and deconvolved using the iterative Richardson–Lucy algorithm with measured point spread functions. Cross-reactivity was corrected in image protocols where a primary antibody reacted with two secondary antibodies. The cross-reactivity was characterized by colocalization of Cy3-associated and α-sarcomeric actinin–associated signal and removed by subtraction of Cy3-associated intensities. Individual myocytes were segmented using a manual deformable triangle mesh fitted in three image planes (XY, XZ, and YZ) using the WGA, α-sarcomeric actinin, Cx43, and DAPI image data.^34,35,41^ The manual segmentation was refined using the WGA image data. A principal component analysis was performed for each segmented myocyte to yield eigenvectors e_1_, e_2_, and e_3_. A bounding box was created for each segmented myocyte using the coordinate system spanned by the eigenvectors. Length, width, and height were determined from the dimensions of the bounding box. Myocyte volume was defined as the volume of voxels within the segmented myocyte, and surface area was estimated from the surface area of the triangle mesh.

### Cx43 analysis

**Figure 2.**
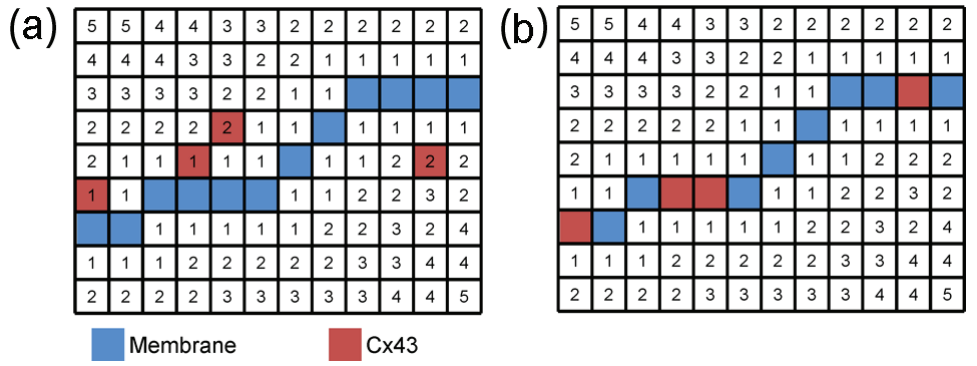
Simplified schematic representation for calculating percentage of membrane positive for Cx43. Voxels are represented on a grid with different colours indicating membrane and Cx43 staining. The integer values represent distance in voxels from the membrane. Gradient vectors were calculated from the distance map, and Cx43 intensities were mapped to the membrane as shown in (b). Cx43: connexin-43.

The percentage of the membrane stained positive with Cx43 was calculated for each segmented myocyte using projections of Cx43 intensities onto the myocyte surface. An illustration of this method is shown in Figure 2. The membrane was approximated by surface voxels around the perimeter of the segmented myocyte. A 3D distance map was calculated from both the inside and outside of the membrane. Gradient vectors were calculated from the distance map. Cx43 intensities within 1 µm of the membrane were projected onto the membrane using the calculated distance map and vectors. The percentage of the membrane positive for Cx43 (Mem_Cx43Pos_) was calculated for each myocyte

$${\text{Mem}}_{\text{Cx}43\text{Pos}}=\frac{{\text{nv}}_{\text{Mem},\text{Cx}43>0}}{{\text{nv}}_{\text{Mem}}}$$

with the number of membrane voxels (nv_Mem_) and the number of membrane voxels with nonzero Cx43 intensity (nv_Mem,Cx43>0_).

The spatial distribution of Cx43 was characterized through projections of Cx43 intensities on the eigenvectors of the myocyte.^35^ Profiles were normalized with respect to total intensities, and the range of arguments was transformed to [−1, 1] (i.e. centered with respect to the respective bounding box dimension). For each eigenvector, polarization (Pol_25%_) was characterized through summation of Cx43 intensities from 25% of either end of the myocyte. The minimal polarization (Pol_25%min_), maximum polarization (Pol_25%max_), and sum of Pol_25%min_ and Pol_25%max_ (Pol_25%total_) were reported. Uniform Cx43 distributions for a profile would lead to Pol_25%total_ of 50%. Higher-order statistical moments, skewness (γ_1_) and kurtosis (γ_2_), were calculated for the Cx43 intensity profiles. Skewness and kurtosis are measures of asymmetry and peakedness, respectively. A skewness of zero indicates that intensities are evenly distributed on both sides of the mean, whereas positive and negative values of skewness indicate that intensities are concentrated in the negative (x < 0) and positive (x > 0) domains, respectively. The kurtosis of a normal and uniform distributions is 0 and −1.2, respectively.

### Myocyte volume fraction

The myocyte volume fraction (MVF) was calculated by down-sampling the processed 3D image data for the α-sarcomeric actinin labeling. Original voxels with dimensions of 0.2 µm × 0.2 µm × 0.2 µm were resampled to 1.6 µm × 1.6 µm × 1.6 µm using the maximum value in a 26-voxel neighborhood relation.^42^ This effectively “blurred” the sarcomeres and filled gaps between adjacent z-disks. Histograms of voxel intensities associated with actinin-positive regions were generated, and thresholds were defined as mode intensity minus one standard deviation. Voxels above the threshold were considered actinin positive. MVF was defined as the sum of actinin-positive voxels divided by the sum of all voxels within the image stack.

### Statistical analysis

The data were reported as mean ± standard deviations. Statistical significance was determined with a one-way analysis of variance (ANOVA) for each measure, followed by post hoc Tukey–Kramer tests with an α = 0.05. Where appropriate, F-tests were performed to determine differences in variances with an α = 0.05.

## Results

### Visual inspection of engineered tissue preparations

**Figure 3.**
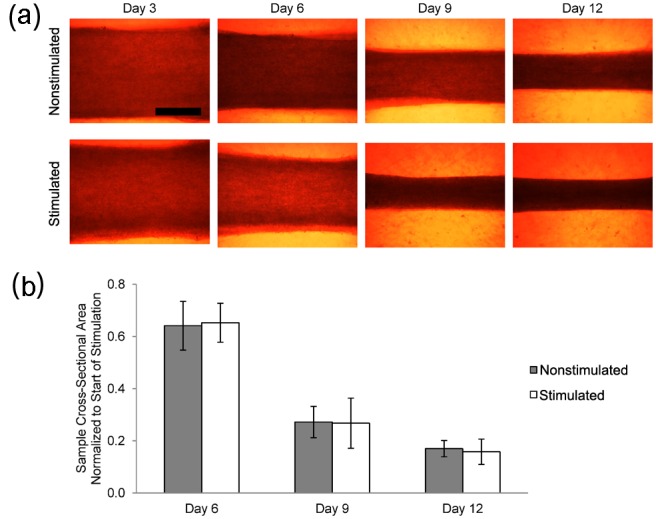
Engineered tissue size. (a) Images showing progression of engineered cardiac tissue size during culture (nonstimulated (n = 9) and stimulated (n = 12)). Scale bar = 1 mm. (b) Cross-sectional area normalized to start of electrical stimulation (day 3). Error bars denote standard deviation. Engineered cardiac tissue progressively decreases in volume as a function of time in culture; however, there is no statistically significant difference between nonstimulated and stimulated engineered cardiac tissues.

**Figure 4.**
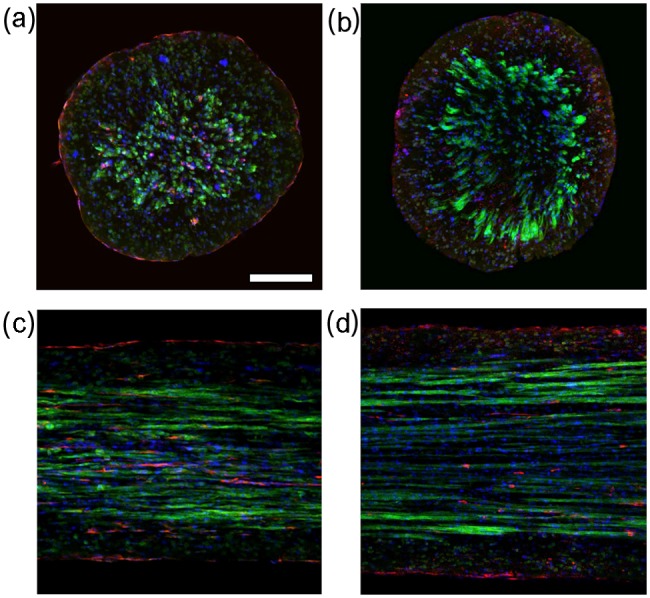
Typical central confocal images of nonstimulated (a and c) and stimulated (b and d) engineered tissue stained with actinin to identify myocytes, vimentin to identify fibroblasts, and DAPI to identify nuclei. (a and b) Transverse and (c and d) longitudinal cross sections. Dense regions of myocytes were found approximately 200 µm from the periphery of the sample. Fibroblasts were found in close spatial proximity to myocytes. Scale bar: (a) 200 µm applies to all. DAPI: 4′,6-diamidino-2-phenylindole dihydrochloride.

**Figure 5.**
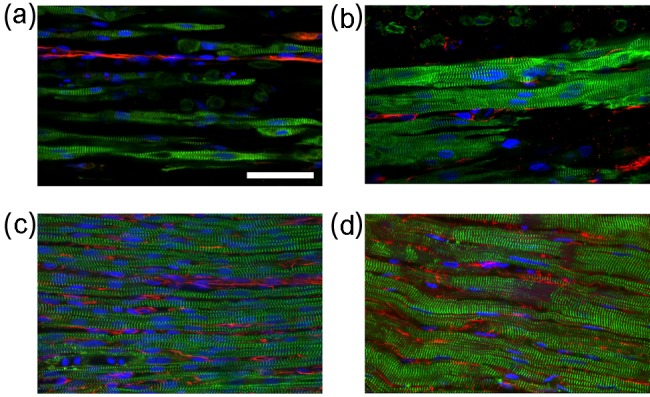
Typical confocal images of cardiac tissue samples stained with actinin to identify myocytes, vimentin to identify fibroblasts, and DAPI to identify nuclei. Engineered tissue from (a) nonstimulated and (b) stimulated samples cultured for 12 days. Left ventricular myocardium from (c) P12 and (d) adult rats. Myocytes and fibroblasts are in close spatial proximity. Visually, the (b) stimulated sample has more densely packed myocytes compared to the (a) nonstimulated sample. P12 and adult rat myocardium have densely packed myocytes and fibroblasts. Scale bar: (a) 50 µm applies to all. DAPI: 4′,6-diamidino-2-phenylindole dihydrochloride.

Bright field images of the engineered tissue samples showed that samples progressively condensed during culture (Figure 3). Engineered tissue sample cross-sectional area estimated from the measured diameter was found to decrease to 17% ± 3% and 16% ± 5% for nonstimulated and stimulated samples at the end of culture from the onset of stimulation. No significant differences in cross-sectional area were observed between the nonstimulated and stimulated samples. Central transverse and longitudinal cross sections of whole tissue samples exhibited dense regions of aligned myocytes and fibroblasts (Figure 4). Although nuclei appeared to be homogeneously distributed through the sample thickness, elongated myocytes were located approximately 200 µm from the sample periphery. Higher magnification confocal images showed that fibroblasts were in close spatial proximity to myocytes; however, P12 and adult native tissue samples exhibited a higher density of fibroblasts and myocytes (Figure 5).

### Functional analysis

**Figure 6.**
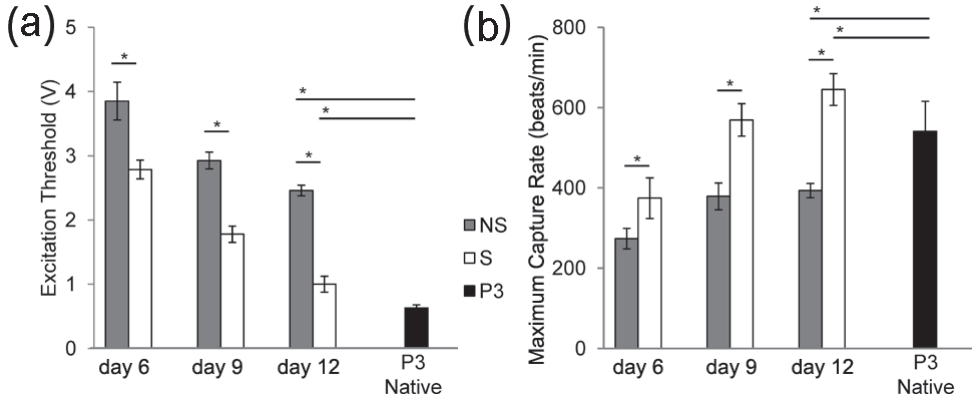
(a) Excitation threshold and (b) maximum capture rate at days 6, 9, and 12 of culture and P3 rat hearts. Excitation threshold progressively decreased and maximum capture rate progressively increased as a function of time in culture. The symbol "*" denotes statistical difference in means determined by post hoc Tukey–Kramer tests (α = 0.05). NS: nonstimulated; S: stimulated; P3: postnatal day 3.

ET and MCR were measured at days 6, 9, and 12 of culture and for isolated strips of P3 left ventricular myocardium. ET and MCR were not measurable at day 3 of culture as the samples did not respond to pacing. The ET decreased as a function of time in culture for both nonstimulated and stimulated samples, and the stimulated samples nearly approached the ET of P3 rat myocardium (Figure 6). Stimulated samples had significantly lower ET at days 6 (2.79 ± 0.15 vs 3.85 ± 0.29 V/cm), 9 (1.78 ± 0.13 vs 2.93 ± 0.13 V/cm), and 12 (1.00 ± 0.12 vs 2.46 ± 0.08 V/cm) of culture compared to nonstimulated samples (p < 0.01). MCR increased as a function of time in the culture for the stimulated group and exceeded that of P3 native myocardium by the end of culture (p < 0.01). Nonstimulated samples exhibited an increase in MCR between days 6 and 9 (p < 0.01) but not between days 9 and 12 (p > 0.05). Furthermore, the stimulated samples had significantly higher MCR at days 6 (374 ± 51 vs 273 ± 25 beats/min), 9 (569 ± 40 vs 379 ± 33 beats/min), and 12 (645 ± 39 vs 393 ± 18 beats/min) of culture compared to nonstimulated samples (p < 0.01).

### 3D confocal imaging

**Figure 7.**
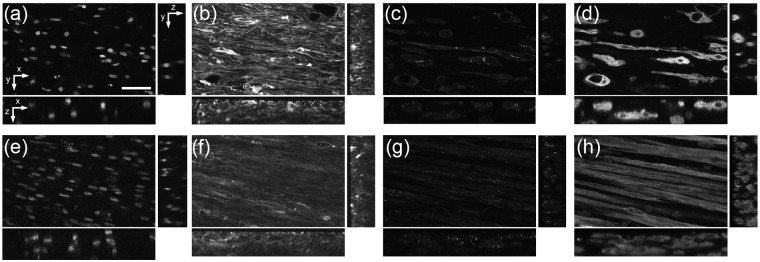
Raw 3D image data of (a–d) nonstimulated and (e–h) stimulated engineered cardiac tissue. (a and e) DAPI, (b and f) WGA, (c and g) Cx43, and (d and h) α-sarcomeric actinin. Scale bar: (a) 50 µm applies to all. 3D: three-dimensional; DAPI: 4′,6-diamidino-2-phenylindole dihydrochloride; WGA: wheat germ agglutinin; Cx43: connexin-43.

The 3D confocal imaging and image analysis were applied to 9 nonstimulated and 12 electrically stimulated engineered tissue constructs and 5 P12 hearts and 7 adult hearts. The approach was applied to preparations stained with WGA, α-sarcomeric actinin, Cx43, and DAPI. Seventy-one image stacks from the four experimental groups were obtained. The image stacks with low SNR or motion artifact were removed from further analysis. Final data were obtained from 7 nonstimulated samples (n = 11 image stacks), 7 stimulated samples (n = 13 stacks), 5 P12 hearts (n = 8 image stacks), and 7 adult hearts (n = 13 image stacks). Raw image data for engineered tissue samples are presented in Figure 7. These stacks originate from ~1 µm outside the tissue surface and extend ~50 µm into the tissue sample.

**Figure 8.**
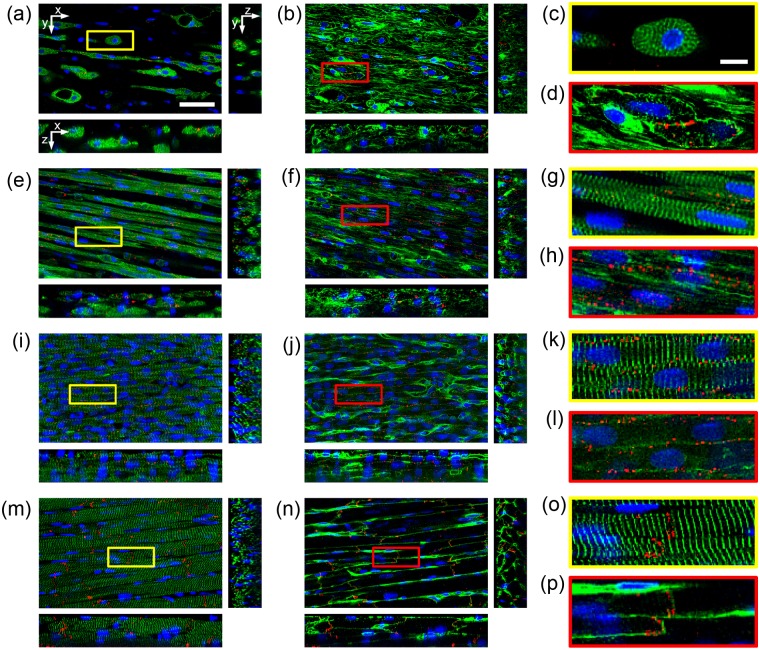
Processed 3D image data of (a–d) nonstimulated engineered cardiac tissue, (e–h) electrically stimulated engineered cardiac tissue, (i–l) P12 native left ventricular cardiac tissue, and (m–p) adult native left ventricular cardiac tissue. (a, e, i, and m) DAPI, α-sarcomeric actinin, and Cx43. (b, f, j, and n) DAPI, WGA, and Cx43. (c, d, g, h, k, l, o, and p) Zoomed regions of (a), (b), (e), (f), (i), (j), (m), and (n), respectively, indicated by box. Scale bar: (a) 50 µm also applies to (b), (e), (f), (i), (j), (m), and (n), and (c) 10 µm also applies to (d), (g), (h), (k), (l), (o), and (p). 3D: three-dimensional; DAPI: 4′,6-diamidino-2-phenylindole dihydrochloride; WGA: wheat germ agglutinin; Cx43: connexin-43.

The processed image stacks from all groups confirmed that myocytes exhibited an elongated morphology (Figure 8). Marked differences between the nonstimulated and stimulated samples were visually noticeable in the 3D image stacks. The stimulated group exhibited more densely packed myocytes with a more pronounced elongated morphology (Figure 8(a) and (e)), aligned sarcomeres in registry (Figure 8(c) and (g)), and more Cx43 plaque formation on the myocyte membrane (Figure 8(d) and (h)). Marked differences between P12 and adult tissues were also apparent by visual observation (Figure 8). P12 myocytes appeared smaller in size (Figure 8(i) and (l)) and had Cx43 plaque formation around the lateral sarcolemma (Figure 8(l)), whereas adult myocytes had Cx43 plaque formation primarily at cell ends (Figure 8(p)).

### MVF

**Figure 9.**
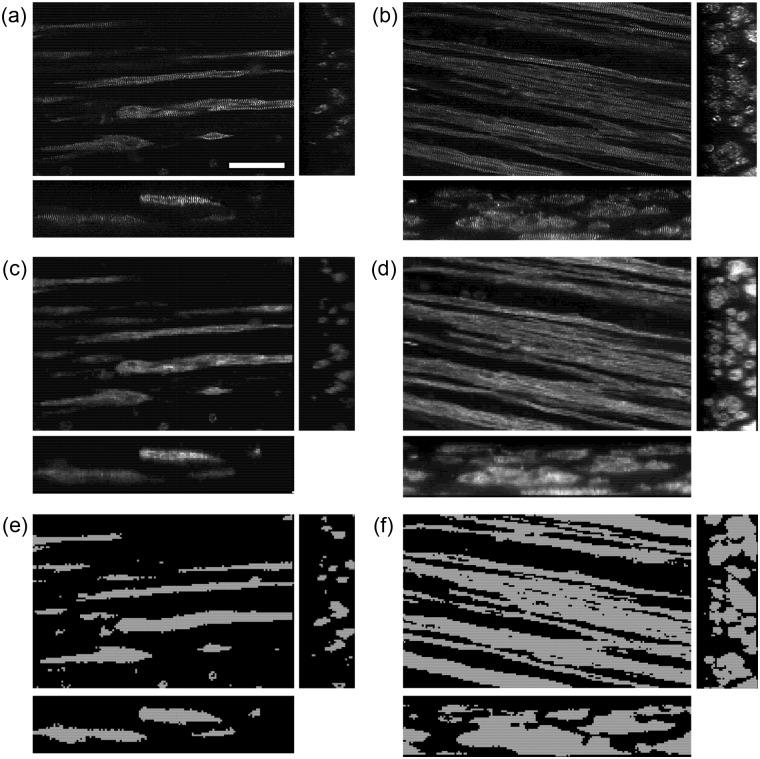
Calculating MVF in the 3D image stacks. (a and b) Actinin labeling is (c and d) resampled at 1.6 µm resolution and (e and f) thresholded to estimate MVF. (a, c, and e) Nonstimulated and (b, d, and f) stimulated. Scale bar: (a) 50 µm applies to (b–f). 3D: three-dimensional; MVF: myocyte volume fraction.

MVF was quantified by down-sampling the processed 3D image data for the α-sarcomeric actinin labeling (Figure 9). Down-sampling of the original images (Figure 9(a) and (b)) resulted in “blurring” of the actinin-associated intensities (Figure 9(c) and (d)). Thresholding of the down-sampled images resulted in identification of the intracellular space of myocytes (Figure 9(e) and (f)). The MVF was nearly double for the stimulated engineered tissue compared to the nonstimulated engineered tissue (0.34 ± 0.14 vs 0.18 ± 0.06, p < 0.01). However, the MVF for both nonstimulated (0.18 ± 0.06) and stimulated (0.34 ± 0.14) engineered tissue was significantly lower than that of P12 (0.90 ± 0.06) and adult (0.91 ± 0.04) myocardium (p < 0.01).

### Myocyte segmentation and Cx43 analysis

**Figure 10.**
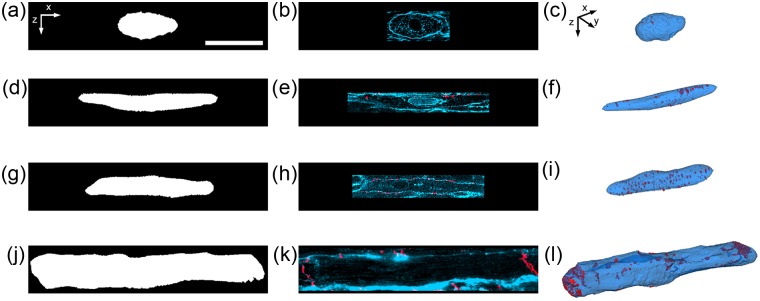
Myocyte segmentation and visualization. (a–c) Nonstimulated, (d–f) stimulated, (g–i) P12 rat, and (j–l) adult rat. (a, d, g, and j) Central XZ-section of segmented myocyte from the image stack. (b, e, h, and k) Corresponding central XZ-section of the image stack with WGA and Cx43. (c, f, i, l) 3D visualization of segmented cells and Cx43. Scale bar: (a) 30 µm applies to all. 3D: three-dimensional; WGA: wheat germ agglutinin; Cx43: connexin-43; P12: postnatal day 12.

Myocyte geometry was quantified through segmentation of individual cells from the 3D image stacks. The segmentation process is shown in Figure 10 with example myocytes from the four experimental groups. The manual manipulation of 3D triangle meshes and thresholding of the WGA channel were used to create 3D reconstructions of myocytes. Central cross sections of the reconstructed myocytes (Figure 10(a), (d), (g), and (j)) served for masking the WGA and Cx43 image data (Figure 10(b), (e), (h), and (k)). The 3D visualizations of the segmented myocytes and associated Cx43 labeling are shown in Figure 10(c), (f), (i), and (l). Myocyte geometry was calculated from the segmented cells (Table 1). Adult myocytes were significantly larger in length, width, height, surface area, and volume compared to nonstimulated and stimulated engineered tissue and P12 native rat myocardium. Length, width, height, surface area, and volume were not statistically different between myocytes from electrically stimulated tissue samples and P12 native myocardium. However, nonstimulated myocytes had more often a rounded morphology as indicated by a smaller mean length compared to stimulated and P12 myocytes and higher widths and heights compared to stimulated myocytes.

**Table 1.** Myocyte geometry and volume fraction.

	Length (µm)	Width (µm)	Height (µm)	Volume (µm^3^)	Surface area (µm^2^)	Myocyte volume fraction
NS: samples, n = 7; segmented cells, n = 64	58.8 ± 21.8^a,b,c^	13.0 ± 2.7^a,c^	10.1 ± 2.5^a,c^	2647 ± 790^c^	1400 ± 381^c^	0.18 ± 0.06^a,b,c^
S: samples, n = 7; segmented cells, n = 58	81.5 ± 19.7^c^	11.3 ± 2.0^c^	8.6 ± 1.6^c^	2968 ± 1296^c^	1775 ± 585^c^	0.34 ± 0.14^b,c^
P12: rats, n = 5; segmented cells, n = 41	72.0 ± 10.9^c^	11.5 ± 1.6^c^	9.1 ± 1.3^c^	3167 ± 783^c^	1732 ± 344^c^	0.90 ± 0.06
Adult: rats, n = 7; segmented cells, n = 51	120.1 ± 31.3	29.4 ± 5.9	19.6 ± 3.3	26,916 ± 11,550	7431 ± 2555	0.91 ± 0.04

NS: nonstimulated; S: stimulated; P12: postnatal day 12.

Values shown are mean ± standard deviation. Statistical difference in means determined by post hoc Tukey–Kramer tests (α = 0.05).

a Difference from S.

b Difference from P12.

c Difference from adult.

**Figure 11.**
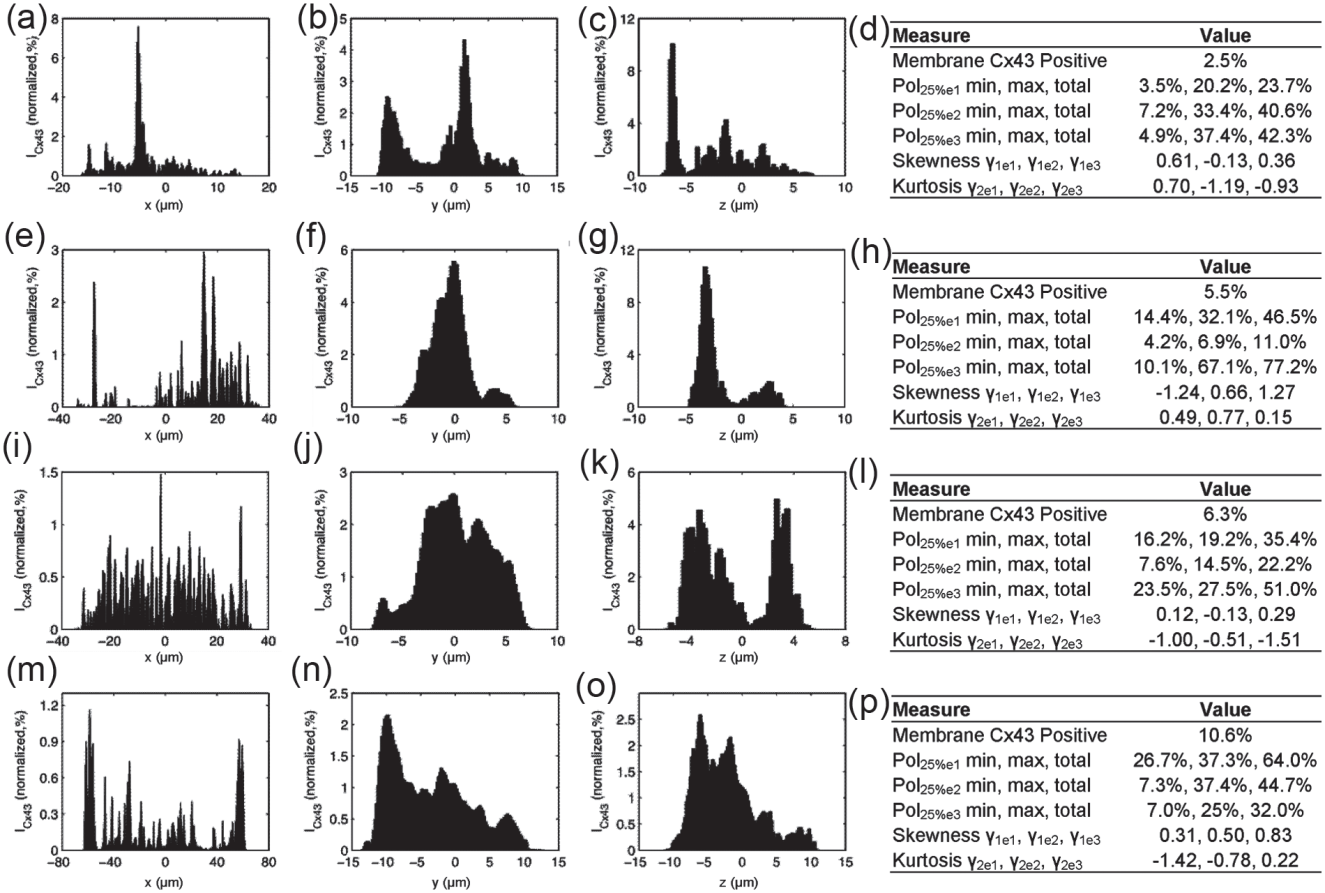
Cx43 intensity profiles from cells in Figure 10. (a–d) Nonstimulated, (e–h) stimulated, (i–l) P12 rat, and (m–p) adult rat. Profiles were produced by projection of Cx43 intensities on the principal axes (a, e, i, and m) e_1_, (b, f, j, and n) e_2_ and (c, g, k, and o) e_3_. Respective quantitative results from example cells are shown in (d), (h), (l), and (p). Cx43: connexin-43; P12: postnatal day 12.

The spatial distribution of Cx43 was characterized through projections of Cx43 intensities on myocyte eigenvectors e_1_, e_2_, and e_3_ and measures of polarization and higher-order statistical moments. Figure 11 shows the profile projections for the segmented example cells in Figure 10. In the nonstimulated myocyte, there was little Cx43 plaque formation indicated by the low percent membrane positive for Cx43 (Figure 11(d)), and a large plaque dominated the profiles as indicated by a sharp peak in the Cx43 projection profiles (Figure 11(a) to (c)). The stimulated myocyte had the majority of Cx43 plaque formation on one end of the cell as can be seen in the profile on eigenvector e_1_ (Figure 11(e)) and the large difference between Pol_25%e1min_ and Pol_25%e1max_ and strong negative skewness (γ_1e1_) (Figure 11(h)). The P12 myocyte had an approximately uniform distribution of Cx43 around the lateral membrane as can be seen in the profile for eigenvector e_1_ (Figure 11(i)). The distribution had a skewness (γ_1e1_) near 0 and a kurtosis near −1.2, which indicates a uniform distribution (Figure 11(l)). Furthermore, the profile for eigenvector e_3_ (Figure 11(k)) for the P12 myocyte showed a bimodal distribution, which indicates that Cx43 plaques were concentrated on the lateral sarcolemma as opposed to cell ends as seen in the adult myocyte. The adult myocyte had the majority of Cx43-associated intensities at cell ends, which can be seen from projections for eigenvector e_1_ (Figure 11(m)) and a Pol_25%e1total_ greater than 50%. The Cx43 distribution was weakly asymmetric as indicated by a small difference in Pol_25%e1min_ and Pol_25%e1max_ and a small positive skewness (γ_1e1_).

**Figure 12.**
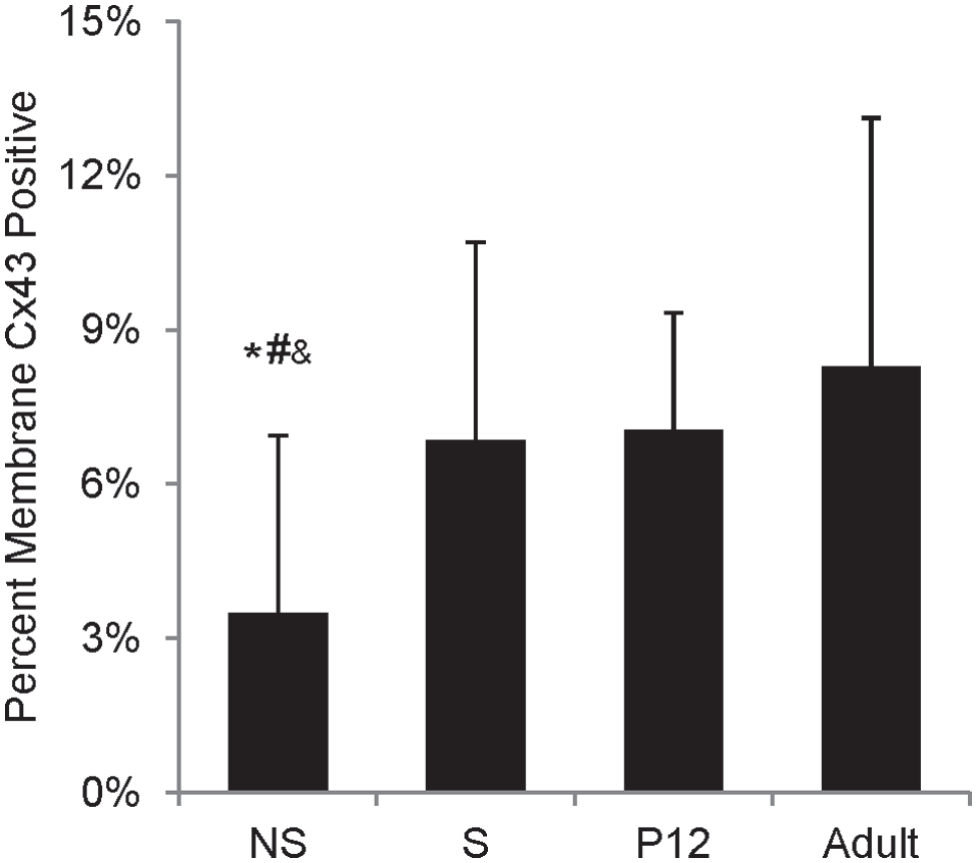
Percentage of membrane stained positive for Cx43. Statistical difference in means determined by post hoc Tukey–Kramer tests (α = 0.05). Symbols denote difference from *S, ^#^P12, and ^&^adult. NS: nonstimulated; S: stimulated; Cx43: connexin-43; P12: postnatal day 12.

The extent of Cx43 plaque formation was assessed by calculating the percentage of membrane positive for Cx43 staining on the segmented myocytes. Nonstimulated engineered tissue had a significantly lower percentage of the membrane area stained positive for Cx43 (3.5% ± 3.4%) compared to stimulated engineered tissue (6.9% ± 3.8%) and that of P12 (7.1% ± 2.3%) and adult (8.3% ± 4.8%) rat myocardium (Figure 12) (p < 0.01).

**Figure 13.**
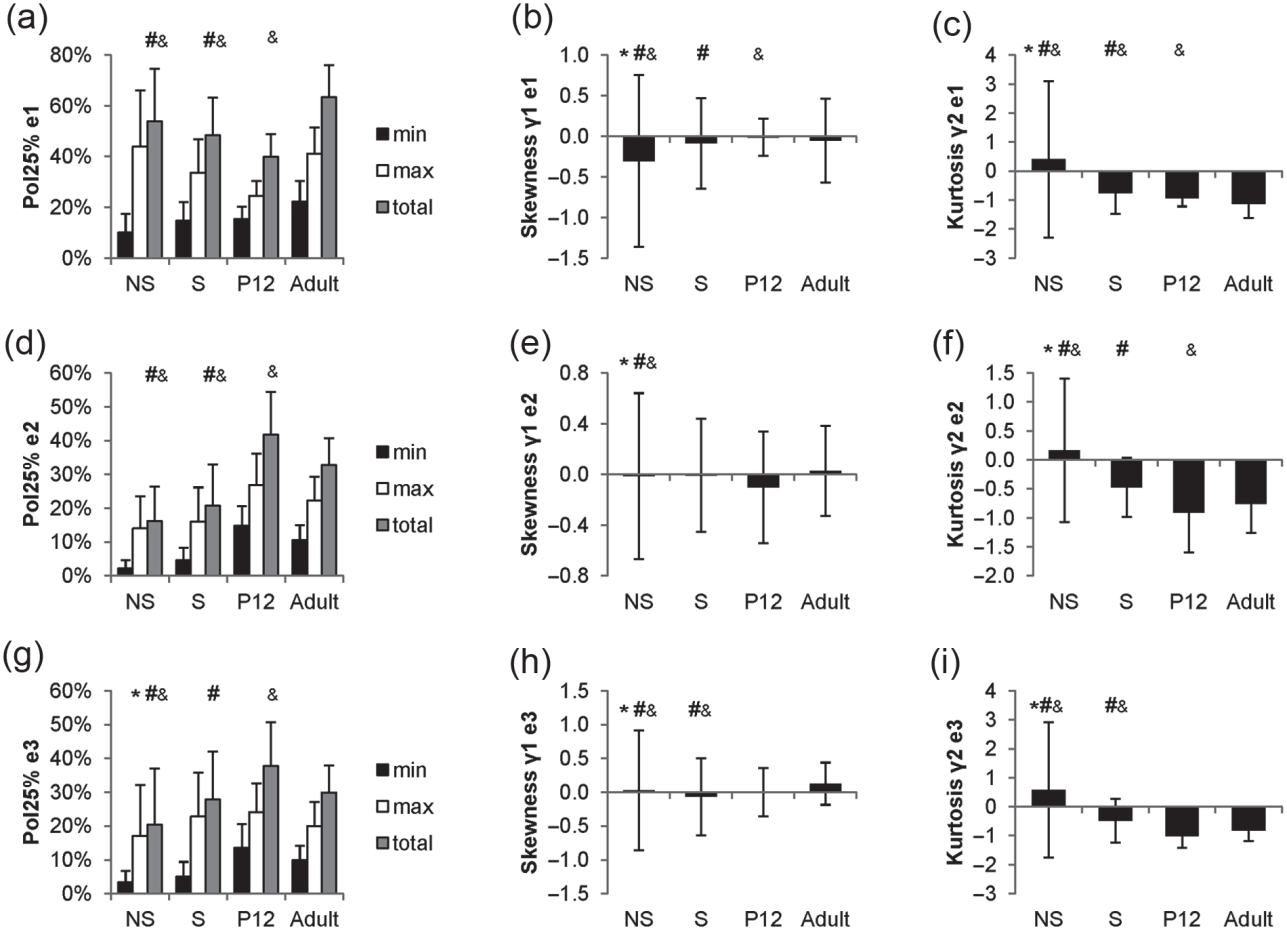
Quantitative results of Cx43 analysis. (a, d, and g) Polarization (Pol_25%_), (b, e, and h) skewness, (c, f, and i) and kurtosis for principal axes (a–c) e_1_, (d–f) e_2_, (g–i) and e_3_. Statistical difference in means of total Cx43 (a, d, and g) polarization (Pol_25%_) determined by post hoc Tukey–Kramer tests (α = 0.05) and variance of (b, e, and h) skewness and (c, f, and i) kurtosis determined by F-tests (α = 0.05). Symbols denote difference from *S, ^#^P12, and ^&^adult. NS: nonstimulated; S: stimulated; Cx43: connexin-43; P12: postnatal day 12.

Statistical results of Cx43 profiles for all segmented cells are presented in Figure 13. Myocytes from nonstimulated and stimulated engineered tissue and P12 native myocardium exhibited no polarization of Cx43, whereas adult myocytes had the majority of their Cx43 concentrated at cell ends (Figure 13(a)). Nonstimulated myocytes had a large difference in Pol_25%e1min_ and Pol_25%e1max_ (Figure 13(a)) and a high standard deviation of skewness (Figure 13(b)), indicating that most cells had Cx43 plaques concentrated on one side of the myocyte. Furthermore, the measured skewness (Figure 13(b), (e), and (h)) and kurtosis (Figure 13(c), (f), and (i)) were highly variable for the nonstimulated group compared to all other groups for all three eigenvector profiles.

## Discussion

In this study, we applied 3D confocal imaging and analysis to test the hypothesis that environmental cues direct engineered tissue toward a phenotype resembling that of age-matched native myocardium. We characterized effects of electrical stimulation on myocyte geometry and the spatial distribution of Cx43 in engineered cardiac tissue, and applied the same techniques for characterization to age-matched and adult native tissue. The results of the study support the hypothesis that electrical stimulation directs engineered cardiac tissue toward a phenotype resembling that of age-matched native myocardium. Adult myocytes were found to have significantly different geometries and Cx43 distributions compared to both engineered tissue constructs and P12 myocardium (Table 1 and Figures 12 and 13). This suggests that age-matched native tissue serves as a more realistic target for both analytical comparison and development of design specifications for engineered tissue samples.

Although other studies have elucidated some of the effects of electrical stimulation on the development of engineered cardiac tissue,^22,28,29^ a novelty of our study is in the comprehensive 3D imaging and analysis approach and quantitative comparison to native tissue. A key feature of the approach is the ability to extract individual myocytes from the image data and assign a reliable coordinate system from which measures of geometry and Cx43 are computed. The common approach of 2D imaging produces a single cross section through a cell, which increases the chances of misinterpreting or overlooking data and introducing variability.

The approach for fabricating tissue samples was based on methods described by Hansen et al.^38^ and was selected because of its success in producing tissue samples with densely packed, aligned myocytes. Both nonstimulated and stimulated engineered tissue samples showed dense regions of myocytes and fibroblasts, the two most abundant cell types in the heart (Figure 4). Our observations of tissue sample development are in agreement with Hansen et al.^38^ Samples condensed to approximately 20% of their initial cross-sectional area (Figure 3), and this process was independent of electrical stimulation. Cells appeared to have a rounded morphology and appeared to be homogeneously distributed through the sample at the beginning of culture. After 3–6 days in culture, the cells began to elongate, align, and contract in isolated regions of the sample. Contracting regions increased in size between 6 and 9 days of culture, and whole samples were macroscopically observed to contract by the end of culture (day 12).

In this study, electrical stimulation had a high impact on MVF. Although the MVF for the electrically stimulated group was less than half of that of P12 and adult myocardium, it was nearly double compared to nonstimulated samples. We suggest that the increase in MVF in the electrically stimulated group is caused by more myocytes developing and maturing. This notion is supported by the fact that both the nonstimulated and stimulated samples started with the same number of cells, construct sizes were not statistically different at the end of culture (Figure 3), and myocytes did not differ in volume (Table 1).

Myocytes subjected to electrical stimulation had geometries and Cx43 distributions that more closely matched P12 myocardium compared to nonstimulated myocytes and native adult myocytes. Under electrical stimulation, myocytes were found to assume an elongated morphology as opposed to their nonstimulated counterparts, which often had a rounded morphology. This is consistent with previous studies.^22^ Furthermore, sarcomeres were often disorganized in nonstimulated myocytes (Figure 8(c)) compared to stimulated samples, P12, and adult tissue (Figure 8(g), (k), and (o), respectively). Moreover, electrical stimulation was found to increase the percentage of membrane positive for Cx43 plaques compared to nonstimulated samples (Figure 12). In fact, the percentage of membrane positive for Cx43 in the electrically stimulated group was not statistically different from P12 or adult rat myocardium.

Previous studies have reported the spatiotemporal dynamics of Cx43 in postnatal development of rat^18^ and human cardiac tissue.^19^ In neonatal rat cardiac tissue, Cx43 plaques were found to be uniformly distributed over the myocyte membrane and remodeled to become concentrated at cell ends (i.e. polarization) at approximately 90 days. In adult rat myocytes, Cx43 is mostly located at cell ends, which was found in our previous^35^ and other studies.^15,43^ Thus, our findings in this study are in agreement as P12 myocytes had no preference for Cx43 plaques at cell ends (Pol_25%_ = 40% ± 9%) compared to adult myocytes, which showed that the majority of Cx43 plaques were found at cell ends (Pol_25%_ = 63% ± 13%). Myocytes from both engineered tissue groups were similar to P12 myocytes and had no significant Cx43 polarization. The Cx43 profiles showed that myocytes from all groups had significant variability with respect to symmetry and polarization. Projections of Cx43 intensities on eigenvector e_1_ indicated that the nonstimulated myocytes had the largest difference between Pol_25%min_ and Pol_25%max_, which was reflected in the large standard deviation of the skewness. Projections on eigenvectors e_2_ and e_3_ showed that the P12 myocytes had significant polarization, indicating that Cx43 plaques were located along the lateral sarcolemma as opposed to centralized regions of cell ends as in adult tissue. The nonstimulated myocytes had the highest standard deviation in both measures of skewness and kurtosis for intensity profiles on all axes, suggesting high variability with respect to Cx43 distributions.

Functional measures of ET and MCR were found to be influenced by electrical stimulation (Figure 6). The lower ET and higher MCR found in the electrically stimulated group are in agreement with other studies, which have applied electrical stimulation.^22,29,33^ The measured ET of 0.63 ± 0.05 V/cm and MCR of 541 ± 75 beats/min for P3 neonatal ventricles measured in this study were in close agreement with other studies, which ranged from 0.74 ± 0.2 to 1.6 ± 0.1 V/cm for ET and 413 ± 7 to 475 ± 25 beats/min for MCR.^33,44^

### Applications of developed approaches

An application of the developed approach is to define specifications for tissue engineering. Specifications are paramount to the engineering paradigm, and tissue engineering is no exception. In this study, specifications were derived from normal age-matched and adult left ventricular myocardium of rat since a central goal of tissue engineering is reestablishing features of the native myocardium. However, specifications can be derived for any requirement, for example, diseased cardiac tissue where the focus may be to understand the effects of pharmaceutical agents.^1,38^

Another application of the imaging approach and analysis is to characterize structural features of stem cell–based engineered tissue samples. Induced pluripotent human stem cells have the potential to differentiate into any cell type^45,46^ and have been specifically differentiated to cardiomyocytes.^47^ However, their application in developing 3D tissue constructs is in its infancy.^48^

The imaging approach can also be applied to characterize other structural features of cardiac tissue. Sarcomeric actinin staining revealed that many myocytes in the nonstimulated group had sarcomeres that appeared to be disorganized compared to the stimulated, P12 and adult myocytes, which had well-defined sarcomeres in registry. Fibroblasts, the majority of cells in the heart, play an important role in normal cardiac function such as maintaining the extracellular matrix (ECM), paracrine signaling, and cell–cell communication with myocytes and other fibroblasts.^7,8,49^ Quantifying sarcomere organization and the spatial relationship of myocytes and fibroblast is of interest for future studies. Furthermore, measurements of function could be used in conjunction with the structural features quantified in this study to elucidate the complex structure–function relationships found in cardiac tissue. Moreover, the application of other environmental conditions, such as the combinational effects of electrical and mechanical stimulation, is of interest for future work.

### Limitations

Limitations relating to the preparation of native cardiac tissue are described in our previous study.^35^ Our approach for structural characterization did not determine if the Cx43 labeling resulted in functional gap junctions. Assessment of phosphorylation^50^ and colocalization with N-cadherin^51,52^ can offer insight into the potential functionality but were not performed in this study. Furthermore, the total Cx43 expression was not quantified in this study. Instead measures of the percent membrane positive for Cx43 were characterized, which can serve as indirect measure of Cx43 expression. Furthermore, the phenotype of the fibroblasts found in our engineered tissue constructs was not characterized. Fibroblasts can differentiate into myofibroblasts in culture, and the myofibroblastic phenotype can be present in injured myocardium.^53^ Myofibroblasts are responsible for remodeling the ECM and paracrine signaling; however, their effects on engineered tissue have not been studied.^54^

Functional analyses were limited to measures of ET and MCR. Although these measures are well established in the field,^22,29,33^ other functional measures of, for instance, electrical conduction and excitation–contraction coupling, would be beneficial for comprehensive assessment of tissue constructs. However, the focus of this study was on characterizing structure through 3D confocal imaging and not on functional analyses. Measures of cardiac structure are not limited to myocyte geometry and Cx43 distributions. However, those were selected because they are known to influence functional properties and undergo significant changes during development and diseased states.^11–13^

Some of our image data required cross-talk correction, which is an a posteriori method based on detailed investigation of signal intensities. Images costained with α-sarcomeric actinin and vimentin exhibited cross-reactivity of vimentin secondary (Cy3) with α-sarcomeric actinin primary, that is, actinin exhibited both Alexa Fluor 633 and Cy3 fluorophores. The cross-reactivity is due to the same species and isotype (mouse IgG_1_) of the antibodies. Vimentin antibodies raised in different species do exist and were tried in this study (e.g. anti-vimentin, C-terminal antibody produced in rabbit; SAB4503083; Sigma–Aldrich), however, without success.

The described method for myocyte segmentation requires 3D confocal imaging and manual manipulation of triangle meshes, both of which are inherently time-consuming and tedious. Automated methods for image acquisition and myocyte segmentation are a possible solution to this issue and will be addressed in future work. A further limitation of the presented approach is related to the volume of imaged regions. Each image stack spans approximately 200 µm × 150 µm × 50 µm of the sample volume. To overcome the relatively small volume of the image stack, several image stacks were obtained, and only regions dense with myocytes were imaged. Dense regions were identified by scanning the sample with a 10× objective lens.
